# Prognostic Value of the Pace of Tumor Progression as Assessed by Serial ^18^F-FDG PET/CT Scan and Liquid Biopsy in Refractory Colorectal Cancer: The CORIOLAN Trial

**DOI:** 10.3390/cancers12102752

**Published:** 2020-09-24

**Authors:** Silvia Camera, Tugba Akin Telli, Erwin Woff, Caroline Vandeputte, Pashalina Kehagias, Thomas Guiot, Gabriela Critchi, Yacine Wissam, Giacomo Bregni, Elena Trevisi, Andrea Pretta, Chiara Senti, Sophia Leduc, Paraskevas Gkolfakis, Frédéric Hoerner, Françoise Rothé, Francesco Sclafani, Patrick Flamen, Amelie Deleporte, Alain Hendlisz

**Affiliations:** 1 Department of Medical Oncology, Institut Jules Bordet-Université Libre de Bruxelles (ULB), 1000 Brussels, Belgium; silvia.camera@bordet.be (S.C.); akintelli.tugba@bordet.be (T.A.T.); yacine.wissam@bordet.be (Y.W.); giacomo.bregni@bordet.be (G.B.); elena.trevisi@bordet.be (E.T.); andrea.pretta@bordet.be (A.P.); paraskevas.gkolfakis@bordet.be (P.G.); amelie.deleporte@bordet.be (A.D.); alain.hendlisz@bordet.be (A.H.); 2 Department of Nuclear Medicine, Institut Jules Bordet-Université Libre de Bruxelles (ULB), 1000 Brussels, Belgium; erwin.woff@bordet.be (E.W.); thomas.guiot@bordet.be (T.G.); gabriela.critchi@bordet.be (G.C.); patrick.flamen@bordet.be (P.F.); 3 GUTS lab, Department of Medical Oncology, Institut Jules Bordet-Université Libre de Bruxelles (ULB), 1000 Brussels, Belgium; caroline.vandeputte@bordet.be (C.V.); pashalina.kehagias@bordet.be (P.K.); chiara.senti@bordet.be (C.S.); sophia.leduc@bordet.be (S.L.); 4 Clinical Trial Conduct Unit (CTCU), Department of Medical Oncology, Institut Jules Bordet-Université Libre de Bruxelles (ULB), 1000 Brussels, Belgium; frederic.hoerner@bordet.be; 5 Breast cancer translational research laboratory, Department of Medical Oncology, Institut Jules Bordet-Université Libre de Bruxelles (ULB), 1000 Brussels, Belgium; francoise.rothe@bordet.be

**Keywords:** pace of progression, colorectal cancer, whole-body metabolically active tumor volume, circulating tumor DNA, circulating tumor cells

## Abstract

**Simple Summary:**

Management of chemorefractory colorectal cancer patient is challenging, and reliable tools which can predict individual patient prognosis and help the decision making are needed. In this study, we hypothesized that the natural pace of cancer growth and progression, as assessed by early changes of a number of imaging and circulating biomarkers which are surrogates of tumor burden (i.e., metabolically active tumor volume, carcinoembryonic antigen, circulating tumor cells and circulating tumor DNA), could predict patient prognosis. By prospectively recruiting 47 eligible patients who had measurements of these biomarkers taken two weeks apart in the absence of any active anti-cancer treatment, we failed to demonstrate our hypothesis. On the other hand, we found that baseline assessment of the same biomarkers was associated with survival outcomes. Larger studies are needed to confirm these findings and translate them into applications for clinical practice.

**Abstract:**

Introduction: Decision making in refractory colorectal cancer (rCRC) is challenging, with limited data available to predict patient outcome. We conducted a study to assess the pace of cancer progression as a potential prognostic and decision tool. Methods: CORIOLAN was a prospective, single-center, single-arm trial recruiting refractory CRC patients with an ECOG performance status of ≤1 and an estimated life expectancy of ≥12 weeks. 18 fluorodeoxyglucose positron emission tomography/computed tomography (^18^F-FDG PET/CT) scan and blood sample collection were carried out at baseline and after 2 weeks with no cancer treatment given between these timepoints. The primary objective was to evaluate the association between pace of cancer progression as defined by changes of the whole-body metabolically active tumor volume (WB-MATV) and overall survival (OS). Exploratory objectives included evaluation of the prognostic value of circulating cell-free DNA (cfDNA), circulating tumor cells (CTCs) and carcinoembryonic antigen (CEA). Results: 47 eligible patients who had received a median number of 5 (range 2–8) prior treatments were enrolled. At the time of analysis, 45 deaths had occurred, with 26% of patients dying within 12 weeks. The median OS was 6.3 months (range 0.4–14.3). The median relative delta between WB-MATV at baseline and 2 weeks was +21%. Changes of WB-MATV, however, failed to predict OS (hazard ratio (HR) 1.3, *p* = 0.383). Similarly, no association was observed between changes of any of the circulating biomarkers investigated and prognosis. By contrast, high WB-MATV (4.2 versus 9.4 months; HR 3.1, *p* = 0.003), high CEA (4.4 versus 7.0 months; HR 1.9, *p* = 0.053), high cfDNA (4.7 versus 7.0 months; HR 2.2, *p* = 0.015) and high CTC count (3.3 versus 7.5 months; HR 6.5, *p* < 0.001) at baseline were associated with worse OS. Conclusions: In this study, approximately 1 out of 4 refractory CRC patients who were judged to have a life expectancy >12 weeks actually died within 12 weeks. Baseline assessment of WB-MATV, cfDNA, CTCs and CEA, but not early change evaluation of the same, may help to refine patient prognostication and guide management decisions.

## 1. Introduction

Over the past decades, slow but meaningful progress has been made in the systemic treatment of colorectal cancer (CRC). In recent clinical trials, the median overall survival (OS) of patients with unresectable disease has consistently reached the 30-month landmark [1,2]. Beyond improvement of supportive measures, more frequent use of organ-directed therapies and implementation of molecular-based treatment selection strategies, much of this progress is due to the increased availability of active cancer drugs [3].

Nevertheless, the number of systemic therapies for advanced CRC patients is still limited. Furthermore, in real-world practice only <40% and <20% of those who progress after a first-line treatment are treated with a third- and fourth-line of therapy, respectively [4,5]. Such a dramatic drop in the proportion of chemo-refractory patients who are suitable for further treatment is paired with a relatively unfavorable risk- and cost-benefit ratio of standard therapies, with marginal median survival advantages coming at the price of substantial clinical and financial toxicity [4,5,6,7]. An alternative treatment option in this setting is enrollment into clinical trials but this can be proposed only to a minority of patients who meet the stringent study eligibility criteria.

As a result, decision making in this setting has increasingly been puzzling. Balancing patient expectations regarding availability and efficacy of further treatments with the need to avoid futile and toxic therapies and preserve a reasonable quality of life is an everyday challenge in clinical practice. In this regard, relying on objective criteria which may reveal individual patient prognosis could help physicians in the optimization of management decisions. Recently, a nomogram has been built to estimate death probability within 12 weeks for refractory CRC patients receiving further treatment [8]. While undoubtedly useful, this nomogram is based on the evaluation of well-established baseline prognostic variables which inform on the patient and disease status at a certain time point. It remains unknown whether the assessment of dynamic parameters capturing the pace of tumor growth and progression in the absence of any active treatment could provide better or complementary information, which may ultimately refine prognostication.

In this study, we have investigated variations of tumor burden, as assessed by different tools, over a 2-week period, and analyzed their association with survival outcomes in a population of refractory CRC patients.

## 2. Patients and Methods

### 2.1. Study Design and Patient Population

CORIOLAN was a single-center, single-arm, prospective, interventional, non-therapeutic study. Eligibility was restricted to patients aged ≥18 years with histologically confirmed, 18 fluorodeoxyglucose positron emission tomography/computed tomography (^18^F-FDG PET/CT) measurable, unresectable advanced CRC who were refractory or intolerant to standard treatments (including fluoropyrimidines, irinotecan, oxaliplatin, monoclonal antibodies (bevacizumab, cetuximab and/or panitumumab) and regorafenib if available). ^18^F-FDG PET/CT target lesions were defined as follows: unequivocal tumor origin, transverse diameter greater than 15 mm on a registered CT image, and an ^18^F-FDG standardized uptake value (SUV) normalized by lean body mass (SUL) higher than 1.5-fold the mean liver SUL + 2× standard deviations (SDs), or in the presence of liver metastasis, 2.0× mean aorta SUL + 2× SD. Patients had to have a life expectancy of >12 weeks, an Eastern Cooperative Oncology Group (ECOG) performance status of ≤1, and normal bone marrow and organ function as defined by a total bilirubin ≤2× upper limit of normal (ULN), AST/ALT/ALP ≤5x upper limit of normal (ULN) and creatinine ≤2× ULN or creatinine clearance >35 mL/min. Main exclusion criteria included administration of chemotherapy, radiotherapy or major surgery within 4 weeks prior to study inclusion, unresolved adverse events from previous treatments, uncontrolled brain metastases, uncontrolled diabetes, or any other active illness or condition that could interfere with study participation.

### 2.2. Study Procedures

After confirmation of eligibility, on day 1 of the study a baseline ^18^F-FDG PET/CT scan, blood tests including carcinoembryonic antigen (CEA), and collection of additional blood samples (2 × 9 mL for whole blood and 2 × 9 mL for plasma extraction) for analysis of other circulating biomarkers were carried out. The same procedures were repeated 2 weeks later (day 15 ± 1). Imaging and circulating biomarkers were assessed by investigators who were blinded to the clinical data. 

No anti-cancer treatment was allowed between day 1 and day 15, while concomitant medications and supportive care measures were administered as needed. After day 15, patient management including further cancer treatment was left to the decision of the treating physician. Patients were followed-up every 2 months for 1 year.

### 2.3. Metabolic Imaging-Based Assessments 

Serial ^18^F-FDG PET/CT scans were performed in strictly identical and standardized conditions following the European Association of Nuclear Medicine (EANM) procedures [9]. The tracer was administered within 60–70 min before starting image acquisition. Patients fasted for 6 h prior the tracer injection (target serum glucose ≤150 mg/dL and <120 mg/dL at the time of ^18^F-FDG administration in diabetic and nondiabetic patients, respectively). No more than 10 min of difference between ^18^F-FDG injection and image acquisition on baseline and 2-week examinations was allowed. A study-specific Standard Procedures Imaging Manual was created and full adherence to the technical specifications outlined in this manual was required. To respect the ^18^F-FDG PET/CT quantifications, a low dose CT was performed to correct the metabolic images. The metabolically active tumor volume (MATV) of a lesion was defined as the volume of tumor tissue demonstrating metabolic activity at or above the aforementioned threshold. Baseline whole-body (WB)-MATV was calculated as the sum of the MATV values of all target lesions, without a predefined limitation on their number. A previously successfully tested cut-off value for WB-MATV (i.e., 100 cm^3^) [10] for day 1 and day 15, while median relative changes for percentage variations on day 15, were used. High tumor metabolic progression index (TMPI) was defined as a relative delta of WB-MATV between day 1 and 15 higher than the median value.

### 2.4. Circulating Free DNA (cfDNA)

Serial blood samples (2 × 9 mL) for plasma extraction were collected in ethylenediaminetetraacetic acid (EDTA) tubes and centrifuged at 2000× *g* for 15 min at 4 °C within 1 h of sample collection to separate the plasma from the peripheral blood cells. Next, plasma samples were stored at −80 °C. Before cfDNA extraction, a second centrifugation step was performed at 10,000× *g* for 10 min. cfDNA was extracted from 3 mL of plasma using the QIAmp circulating nucleic acid kit (Qiagen, Antwerp, Belgium). DNA was quantified using the Qubit® 2.0 fluorometer and the Qubit® dsDNA HS assay kit (Life-Technologies, Gent-Brussels, Belgium). A previously successfully tested cut-off value (i.e., 50 ng/mL) [11] for day 1 and day 15 (which was very close to the median value, i.e., 46.5 ng/mL), while median relative changes for percentage variations on day 15, were used.

### 2.5. Circulating Tumor Cell (CTC) count

Serial blood samples (2 × 9 mL) for CTC analysis were collected in CellSave preservative tubes (Janssen Pharmaceutica N.V., Beerse, Belgium) and saved at room temperature. CTC count was performed within 72 h after blood collection using the US Food and Drug Administration (FDA)-cleared CellSearch® system (Veridex, Raritan, NJ, USA) according to the manufacturer’s instructions. CTCs were defined as epithelial, nucleated cells expressing cytokeratin but not CD45. CTC enumeration was performed using the CellTrack® Analyzer II (Veridex, Raritan, NJ, USA), which is a semi-automated fluorescence-based microscopy system that allows computer-generated reconstruction of cellular images. A previously validated cut-off value (i.e., 3 CTCs) [12] for day 1 and day 15, while median relative changes for percentage variations on day 15, were used.

### 2.6. Carcinoembryonic Antigen (CEA)

Serial CEA was measured according to standard laboratory procedures (ULN 5.2 μg/L). Median values were used as cut-off for both baseline and early variation assessment.

### 2.7. Study Objectives

The primary objective of the study was to assess the prognostic value of the TMPI as measured by variations of WB-MATV on serial ^18^F-FDG PET/CT scans performed at baseline and on day 15. Secondary objectives included evaluation of the prognostic role of baseline factors (including age (<70 versus ≥70 years), sex, body mass index (<25 versus ≥25), ECOG performance status (0 versus 1), tumor sidedness (right-sided versus left-sided), time to refractory disease (<24 versus ≥24 months), number of metastatic sites (<3 versus ≥3), peritoneal involvement (absent versus present), WBC (<10^9^/L versus ≥10^9^/L), neutrophil/lymphocyte ratio (<5 versus ≥5), Hb (<11 g/dL versus ≥11 g/dL), ALP (<300 IU/L versus ≥300 IU/L), LDH (<300 IU/L versus ≥300 IU/L), CRP (median), RAS status (mutant versus wild type), WB-MATV (<100 cm^3^ versus ≥100 cm^3^), and baseline and early variations of liquid biomarkers (CEA, cfDNA, and CTCs). In a post-hoc analysis, the association between the “Colon Life” score as calculated by Pietrantonio et al [8] and OS was assessed.

### 2.8. Statistical Considerations

The primary outcome measure was OS which was measured from day 1 until death from any cause. Patients lost to follow-up were censored at the time of the last contact. Based on our previously published data [13], we estimated that patients with a low TMPI had a 60% reduction in the risk of death as compared with those with a high TMPI. Assuming a median OS of 4 months for the overall population, a hazard ratio (HR) of 0.40 would translate into a median OS of 5.7 and 2.3 months for low and high TMPI patients, respectively. Based on a 2-sided α = 0.05 and a β = 0.20, 47 assessable patients and 37 events were needed. Survival analyses were performed using the Kaplan-Meier method. Cox proportional hazards model was used to estimate the HR with 95% confidence intervals (CIs). Logrank tests were used to compare survival curves. Statistical analyses were performed using R version 3.5.1.

### 2.9. Approvals and Consent

The study was approved by the Institutional Review Board at the Institut Jules Bordet and by an Independent Ethics Committee (CE1911). The study is registered at clinicaltrials.gov (NCT01591590). The trial was conducted according to the principles set in the Declaration of Helsinki. All patients provided a written informed consent before any study procedure was performed.

## 3. Results

**Figure 1:**
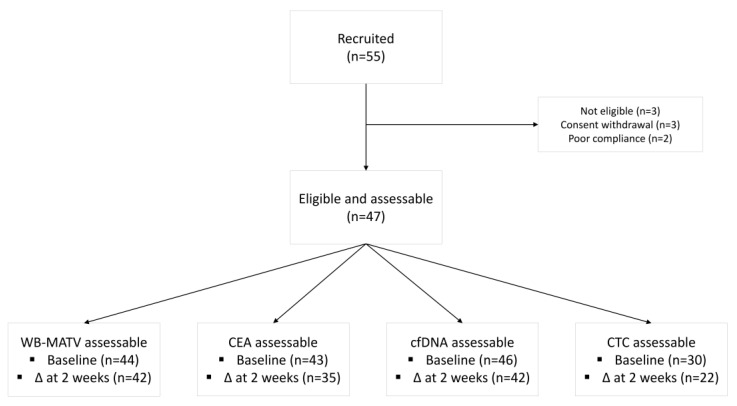
Study flow diagram. Abbreviations: WB-MATV, whole body metabolically active tumor volume; CEA, carcinoembryonic antigen; cfDNA, circulating-free DNA; CTC, circulating tumor cell; Δ, delta.

Between June 2012 and May 2018, 55 patients were enrolled in the study. Of these, 8 were excluded from the analysis due to ineligibility (*n* = 3), withdrawal of the informed consent (*n* = 3) and poor compliance with the study procedures (*n* = 2) (Figure 1). Baseline characteristics of the 47 eligible patients are summarized in Table 1. Median age was 65 years (range 38–82), 64% of patients had an ECOG performance status of 1, and the median number of prior treatments was 5 (range 2–8). Tumors of 57% and 0% of patients were known to harbor *RAS* and *BRAF* mutations, respectively. In line with the study eligibility criteria, no patient received any cancer treatment between day 1 and 15. After day 15, 23 (49%) patients were treated with investigational agents within the context of a phase I clinical trial (*n* = 10), a placebo-controlled clinical trial (*n* = 3), regorafenib (*n* = 8), trifluridine/tipiracil (*n* = 1) and capecitabine (*n* = 1). At the time of analysis, 45 deaths had been observed, of which 12 (26%) occurred within 12 weeks of study entry. The median OS was 6.3 months (range 0.4–4.3).

### 3.1. Prognostic Value of Metabolic Imaging Parameters 

**Figure 2:**
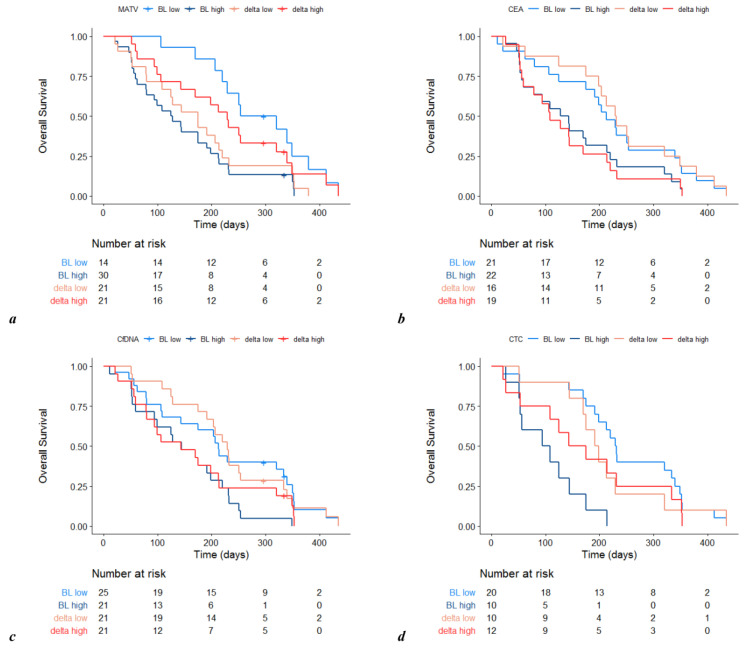
Overall survival by baseline (BL) and early 2-week change (delta) of WB-MATV (**a**), CEA (**b**), cfDNA (**c**) and CTC (**d**). Abbreviations: WB-MATV, whole body metabolically active tumor volume; CEA, carcinoembryonic antigen; cfDNA, circulating-free DNA; CTC, circulating tumor cell. Prognostic value of liquid biomarkers.

An ^18^F-FDG PET/CT scan was carried out on day 1 in 44 (94%) and on day 15 in 42 (89%) patients. High WB-MATV both at baseline (*n* = 30/44, 68%) (4.2 versus 9.4 months; HR 3.1, 95% CI 1.5–6.4, *p* = 0.003) and on day 15 (*n* = 22/42, 52%) (4.7 versus 7.9 months; HR 2.2, 95% CI 1.0–4.6, *p* = 0.044) was associated with a worse OS. In 42 cases (89%), variations of WB-MATV between day 1 and day 15 were assessable. The median relative delta was +21%, and 3/14 (21%) low baseline WB-MATV patients were observed to have high WB-MATV tumors two weeks later. Changes of WB-MATV did not predict OS, patients with high TMPI having similar prognosis to those with low TMPI (7.0 versus 5.7 months; HR 1.3, 95% CI 0.71–2.6, *p* ≥ 0.383) (Figure 2).

Forty-three (91%), 46 (98%) and 30 (64%) patients were assessable for CEA, cfDNA and CTC, respectively, at baseline. High CEA (4.4 versus 7.0 months; HR 1.9, 95% CI 1.0–3.5, *p* = 0.053), high cfDNA (*n* = 21, 46%) (4.7 versus 7.0 months; HR 2.2, 95% CI 1.2–4.3, *p* = 0.015) and a high CTC count (*n* = 10, 33%) (3.3 versus 7.5 months; HR 6.5, 95% CI 2.4–17.0, *p* < 0.001) predicted worse OS. Variations of CEA, cfDNA and CTC count between day 1 and day 15 were assessable for 35 (74%), 42 (89%) and 22 (34%) patients, respectively, with the median relative delta for each of the same biomarkers being +16.3%, +6%, and +100%. In no case, these variations were associated with OS (CEA: HR 1.9, 95% CI 0.94–3.8, *p* = 0.073; cfDNA: HR 1.6, 95% CI 0.86–3.1, *p* = 0.133; CTC count: HR 1.2, 95% CI 0.49–2.9, *p* = 0.703) (Figure 2).

### 3.2. Other Prognostic Factors at Baseline 

Among the other baseline prognostic factors analyzed, low Hb (HR 2.2, 95% CI 1.2–4.0, *p* = 0.017), high neutrophil/lymphocyte ratio (HR 2.4, 95% CI 1.3–4.5, *p* = 0.006), high ALP (HR 3.5, 95% CI 1.7–7.5, *p* < 0.001), and high CRP (HR 3.5, 95% CI 1.8-6.7, *p* < 0.001) predicted poor OS. Forty-three patients (91%) could be scored using to the Colon Life nomogram. The median estimated probability of death at 3 months was 30% (range 10–94%). In patients with lower Colon Life scores, median OS was 6.9 months (range 1.7–12.5 months), while in those with higher Colon Life scores median OS was 4.7 months (range 0.4–14.3 months) (HR 1.2, 95% CI: 0.6–2.2, *p* = 0.648). Receiving further treatment after completion of the trial was associated with a numerically, but not statistically significantly, longer OS (median OS 7.1 versus 3.5 months; HR 0.66, 95% CI 0.36–1.2, *p* = 0.17).

## 4. Discussion

In this study, we have failed to show that taking advantage of a two-week treatment free period to evaluate changes of surrogate markers of tumor burden could be a useful strategy to predict the survival of patients with chemorefractory CRC. 

Outcome prediction in advanced CRC is extremely valuable. Information such as tumor aggressiveness, probability of clinical benefit versus toxicity from cancer treatments, and likely life expectancy with or without treatment can inform the discussion between patient and physician and ultimately help the decision making. Substantial progress has recently been made in this regard, but most of the available data are based on studies conducted in chemonaïve patients. Köhne et al and Renfro et al validated an algorithm and nomograms, respectively, to predict prognosis of CRC patients using data from CRC patients who were treated with first-line therapy [14,15]. It is clear, however, that a number of key patient- and disease-related factors, as well as the actual and perceived relevance of the same, change dramatically along the progression from first- to later lines of therapies, and using the same evidence to inform prognosis and guide management decisions across all treatment settings may not be entirely appropriate.

The design of our study was based on the assumption that natural dynamics of cancer growth and progression over a relatively short time period could be more informative than a snapshot of the burden of cancer at a single time point. To assess this, we analyzed a group of patients that were highly representative of the chemorefractory CRC population with regard to baseline characteristics, treatments received and overall prognosis. Importantly, we used a number of imaging and circulating parameters that, while not routinely tested in clinical practice, have all previously been reported to be reliable markers of tumor burden and indicators of CRC prognosis, with an ever-increasing interest for their potential clinical applications [10,11,12,16]. Despite the sound rationale, however, our hypothesis was not confirmed, the dynamics of any of the tested markers being associated with survival. 

While the negative results of our study appear to refute the general thinking that the pace of progression should be considered as a surrogate of cancer aggressiveness, these may be explained by a number of factors. First of all, it is possible that the time interval used to assess the spontaneous pace of cancer progression (i.e., 2 weeks) was too short to detect clinically relevant variations of the markers analyzed. This interval, however, was pragmatically set and corresponds overall to the washout time window that is generally needed in clinical practice to allow patients to recover from most toxicities from previous treatments and to make all necessary arrangements for the start of a new line of therapy. Holding cancer treatments for longer than two weeks especially in a chemorefractory population would have likely been felt unacceptable by most patients and physicians. Second, as expected, approximately half of the study patients received further post-study cancer treatments. While the survival gain from standard therapies and investigational drugs in this setting is limited, [4,6,17] a direct association between pace of tumor progression and treatment benefit is possible (i.e., patients with rapidly progressing tumors being most likely to respond to treatment), and such an association could have confounded the survival data. Third, the small number of assessable patients and limited statistical power (especially for some of the circulating biomarkers) might have precluded that numerically different survival outcomes translated into statistically significant findings. Fourth, despite our efforts to standardize the methodology of the ^18^F-FDG PET/CT scan and liquid biopsy, it is still possible that some uncontrollable analytical biases affecting the comparison between baseline and the 2-week time point might have occurred. Finally, it cannot be excluded that, despite conceptually valid and clinically intriguing, the study hypothesis was mistakenly set in the first place. The prognostic relevance of the baseline static assessment of tumor burden in the setting of chemorefractory CRC may still be more important than the early variations of the same, an assumption that is actually supported by the results of secondary analyses of our study.

One of the most useful applications of prognostic tools for chemorefractory cancer patients is the selection of potential candidates for clinical trials which generally require a life expectancy of ≥12 weeks. Estimating the survival of individual patients, however, is challenging, and the inaccuracy of life expectancy estimation based on a physician judgement which largely relies on conventional criteria is confirmed by our findings. While eligibility for our study was also based on the assumption that patients would live longer than 12 weeks, one out of 4 patients did not ultimately meet this criterion. Recently, a prognostic nomogram (i.e., Colon Life), which is based on four parameters including ECOG performance status, LDH, surgical resection of the primary tumor and presence of peritoneal metastases, has been proposed to address this issue [8]. Interestingly, this nomogram failed to accurately predict outcome in our patient population. There is no doubt that the lack of reproducibility of the prognostic value of Colon Life in our series can be explained by the relatively small study numbers and the fact that only half of our patients eventually received active treatment. This, however, highlights the need to explore alternative, potentially more informative, prognostic parameters. In this regard, our analysis suggests that, in addition to classical prognostic factors such as low Hb, high neutrophil/lymphocyte ratio, high ALP, and high CRP, baseline evaluation of tumor burden according to more sophisticated surrogate markers such as WB-MATV, cfDNA and CTCs may help to refine prognostication and eventually guide decision regarding trial recruitment.

We acknowledge the limitations of our study. These include the small sample size which reduced the statistical power especially for the circulating biomarkers, precluded running multivariable analyses, and did not allow formal comparisons of the novel biomarkers with conventional prognostic factors. Other limitations are the fluctuating proportions of assessable patients, and the potential risk of random findings due to multiple comparisons. Additionally, it should be noted that study entry was restricted to refractory CRC patients with an ECOG performance status of ≤1 (who are those suitable for phase I clinical trials), this limiting generalizability of our results to the population patients who, while having an ECOG performance status of >1, may be still candidates for further treatment. Nevertheless, the prospective design, the analysis of novel markers and the standardization of analytic procedures make this a unique study which adds substantially to the current knowledge about prognostication in chemorefractory CRC. While requiring confirmation in independent series, our findings provide the basis for the development of future studies which may ultimately shape the decision making in this critical clinical setting.

## 5. Conclusions

In this study, we have failed to show that early changes of imaging and circulating biomarkers over a two-week period without active treatment predict prognosis of patients with chemorefractory CRC. On the other hand, baseline assessment of these biomarkers may help to refine patient prognostication and guide management decisions.

## Figures and Tables

**Table 1:** Baseline patient characteristics.

Variable		N	%
Age (years)(median/range)		65	38–82
Sex	Male	25	53
	Female	22	47
ECOG PS	0	17	36
	1	30	64
Body mass index (kg/m^2^)	≤25	22	47
	>25	25	53
Tumor sidedness	Right	10	21
	Left	33	70
	Unknown	4	9
*RAS* status	Wild type	22	47
	Mutant	25	53
Number prior systemic therapies (median/range)		5	2–8
Number of metastatic sites	≤2	20	43
	>2	27	57
Peritoneal metastases	Absent	33	70
	Present	14	30
Hemoglobin (g/dL)	<11	16	34
	≥11	31	66
NL ratio	<5	28	60
	≥5	18	38
	Unknown	1	2
ALP (IU/L)	<300	34	73
	≥300	10	21
	Unknown	3	6
Colon Life score (%) (median/range)		30%	10–94
WB-MATV (cm^3^)	<100	14	30
	≥100	30	64
	Unknown	3	6
cfDNA (ng/mL)	<50	25	53
	≥50	21	45
	Unknown	1	2
CTC count (/mL)	<3	20	53
	≥3	10	45
	Unknown	17	2

Abbreviations: ECOG PS—Eastern Cooperative Oncology Group Performance Status; NL—neutrophil to lymphocyte; ALP—alkaline phosphatase, WB-MATV—whole body metabolically active tumor volume; cfDNA—circulating-free DNA; CTC—circulating tumor cell.
